# Finite-Blocklength Analysis of Coded Modulation with Retransmission

**DOI:** 10.3390/e26100863

**Published:** 2024-10-14

**Authors:** Ming Jiang, Yi Wang, Fan Ding, Qiushi Xu

**Affiliations:** 1 National Mobile Communications Research Laboratory, Southeast University, Nanjing 210096, China; wang_yi@seu.edu.cn (Y.W.); fan_d@seu.edu.cn (F.D.); xuqiushi@seu.edu.cn (Q.X.); 2 Purple Mountain Laboratories, Nanjing 211100, China

**Keywords:** finite blocklength, retransmission scenario, rate prediction

## Abstract

The rapid developments of 5G and B5G networks have posed higher demands on retransmission in certain scenarios. This article reviews classical finite-length coding performance prediction formulas and proposes rate prediction formulas for coded modulation retransmission scenarios. Specifically, we demonstrate that a recently proposed model for correcting these prediction formulas also exhibits high accuracy in coded modulation retransmissions. To enhance the generality of this model, we introduce a range variable $P_{\mathrm{final}}$ to unify the predictions with different SNRs. Finally, based on simulation results, the article puts forth recommendations specific to retransmission with a high spectral efficiency.

## 1. Introduction

New-generation mobile communication systems, 5G NR networks, are worldwide-deployed communication systems. The 5G wireless system, which is not the straightforward evolution of traditional 4G cellular networks, is developed as a multipurpose mobile network with many new service functionalities [1]. 5G networks can provide not only traditional voice and data communication but also numerous new use cases, applications for various industries, and connectivity for devices and applications across society [2,3]. Examples include vehicle-to-vehicle and vehicle-to-infrastructure communication, industrial automation, health services, smart cities, and smart homes [4,5]. Compared to 4G LTE, 5G NR and the future development of B5G systems have introduced a series of technical indicators. To meet these performance requirements, 5G systems will leverage various emerging technologies, such as heterogeneous networks (HetNets) [6,7], massive multiple-input multiple-output (mMIMO) [8], millimeter-wave (mmWave) communication [9,10], device-to-device (D2D) communication [11,12], machine-to-machine (M2M) communication [13], reconfigurable intelligent surfaces (RISs) [14], and network slicing [15], among others.

D2D and M2M communications have many different characteristics compared to the traditional communication services designed for human interaction. For instance, the communication among many sensors and controllers in closed-loop control systems of automated industries requires a maximum latency of 5 ms and a reliable packet error rate ranging from $10^{-2}$ to $10^{-5}$ [16]. In terms of traffic safety, the packet error rate cannot exceed $10^{-5}$. These typical applications involve short data packets (code length ranges from several hundred to one thousand) and impose very high requirements on latency and reliability. For the applications targeting these machine communications, various solutions have been proposed, including fewer symbols in OFDM signal packets, reducing transmission time. The theoretical limit for the transmission of these short data packets depends on the specific transmission environments and the technologies employed.

Shannon’s limit provides the theoretical maximum performance when the encoding blocklength tends towards infinity. However, in practical situations, Shannon’s limit does not apply to the performance of moderate-length codes [17]. The finite-blocklength performance bounds in binary input additive white Gaussian noise (BIAWGN) channels have adequately addressed this issue. Recently, numerous significant advancements related to finite-blocklength analysis have emerged. Ref. [18] leverages the property of joint convexity to address a broad spectrum of use cases, thereby facilitating the efficient resolution of joint optimization problems in multi-user environments in the finite-blocklength regime. In [19], Behrooz Makki derives closed-form expressions for message-decoding probabilities, throughput, expected delay, and error probability in hybrid automatic repeat request (HARQ) configurations. Moreover, the expectation and variance of the maximum achievable rate in a mMIMO system with a finite blocklength are rigorously analyzed [20]. However, the finite-blocklength performance analysis is not suitable for higher-order modulation schemes, for which effective solutions have been proposed in the existing literature [21,22,23]. Moreover, to ensure transmission reliability while also meeting low-latency constraints, a limited number of retransmissions is typically required in practical wireless networks. Combining rate-compatible coding and incremental redundancy retransmission schemes, the performance analysis of finite-length coded retransmission with high-order modulation is an urgent issue that needs to be addressed.

In this paper, we analyze the performance of finite-length coded modulation in a retransmission scenario when rate-compatible code is modulated and transmitted using different modulation schemes in the first and second transmissions. The remaining structure of this paper is represented as follows. Firstly, we review the theoretical formulas for predicting the performance of finite-length coded modulation and provide a brief explanation of the calculation of key parameters in this formula under retransmission scenarios. Next, we revisit the model for tuning on the theoretical prediction formula and elaborate on the usage of the model. Here, we refine this method to make it more general. Finally, through simulation results, we demonstrate the good adaptability of this calibration model to retransmission scenarios. Based on the simulation results, we also offer some recommendations for the retransmission approach of coded modulation.

## 2. Preliminaries

### 2.1. Some Bounds for Finite-Blocklength Coding

Consider a code with a codebook of size *M* and blocklength *n*, where the rate *R* can be expressed as

(1) $$R=\frac{\log_{2}M}{n}.$$

Building upon this, [24] has proposed formulas for the upper and lower bounds on the performance of finite-length coding. For example, the upper bound like the converse bound, the lower bound like the random coding union (RCU) bound and the dependence testing (DT) bound. However, these bounds all involve a greater amount of summation and combinatorial operations, leading to a higher overall complexity and potentially imprecise results.

For a binary symmetric channel (BSC) with a crossover probability of δ, when it achieves the block error rate (BLER) ϵ, its RCU bound and DT bound can be calculated by

(2) ϵ≤∑t=0nntδt(1−δ)n−tmin{1,(M−1)∑k=0tnk2−n},

and

(3) ϵ≤∑t=0nntmin{δt(1−δ)n−t,(M−1)2−n−1},

respectively. In practical coding, the blocklength *n* usually amounts to several hundred, or even greater than 1000. When the combinations of nk are calculated, the computation process becomes slower, and the precision of the results is certainly affected.

The converse bound of a BSC satisfies

(4) $$M\leq \frac{1}{\beta_{1-\epsilon }^{n}},$$

(5) $$\beta_{\alpha }^{n}=\left(1-\lambda \right)\beta_{L}+\lambda \beta_{L+1},$$

where the $\beta_{l}$ in (5) is defined as

(6) βl=∑k=0lnk2−n,l=L,L+1,

and the integer *L* and variable λ(0≤λ≤1) should be determined by the following equation:

(7) $$\alpha =\left(1-\lambda \right)\alpha_{L}+\lambda \alpha_{L+1},$$

with

(8) αl=∑k=0l−1nk(1−δ)n−kδk,l=L,L+1.

The calculation of the converse bound (4) not only involves the combinatorial operations but also requires solving roots for two parameters in a system of binary equations, making the computation quite complicated. Therefore, a simpler and more efficient calculation method is further explored by normal approximation (NA).

### 2.2. Normal Approximation Combined with Coded Modulation

Given a finite blocklength *n*, BLER ϵ, the upper bound of the rate can be predicted by

(9) $$R=C-\sqrt{\frac{V}{n}}Q^{-1}\left(\epsilon \right)+O\left(\frac{\log_{2}n}{n}\right),$$

which is called normal approximation [24], where $Q\left(x\right)=\int_{x}^{+\infty }\frac{1}{\sqrt{2\pi }}e^{-\frac{1}{2}t^{2}}dt$, *C* and *V* are the channel capacity and the channel dispersion, respectively. They are both characteristic parameters of the channel, where the physical quantities do not depend on the encoding scheme. The third-order term $O\left(\frac{\log_{2}n}{n}\right)$ is proven to be $\frac{\log_{2}n}{2n}$ in [24].

In different channels, *C* and *V* have different calculation methods. For a BSC with a crossover probability of δ and $\delta \notin \{0,\frac{1}{2},1\}$, we have

(10) C=1−h(δ)

(11) $$V=\delta \left(1-\delta \right){\left(\log_{2}\frac{1-\delta }{\delta }\right)}^{2},$$

where $h\left(x\right)=-x\log_{2}x-\left(1-x\right)\log_{2}\left(1-x\right)$.

Meanwhile, for a binary erasure channel (BEC) with an erasure probability of δ, we have

(12) C=1−δ

(13) V=δ(1−δ).

Here, for more general applications, taking a BIAWGN channel with an SNR of *P* into consideration, we have

(14) $$C=\frac{1}{2}\log_{2}\left(1+P\right)$$

(15) $$V=\frac{P}{2}\frac{P+2}{{\left(P+1\right)}^{2}}\log_{2}^{2}e.$$

Although (14) and (15) can be easily calculated, specific modulation methods do not provide the correlation between *C*, *V* and constellations.

If the input *m* points of a constellation, such as *m*-QAM, follow the discrete uniform distribution, the two parameters *C* and *V* can be computed [25] by

(16) $$\begin{array}{c} C_{m}\left(P\right)=\log_{2}m-\frac{1}{m}\sum_{i=1}^{m}E\left[\log_{2}\left(\sum_{j=1}^{m}e^{{∥Z∥}^{2}-{∥x(i)+Z-x(j)∥}^{2}}\right)\right] \end{array}$$

(17) $$\begin{array}{c} V_{m}\left(P\right)=\frac{1}{m}\sum_{i=1}^{m}\mathrm{Var}\left[\log_{2}\left(\sum_{j=1}^{m}e^{{∥Z∥}^{2}-{∥x(i)+Z-x(j)∥}^{2}}\right)\right] \end{array}$$

where *Z* is a complex Gaussian variable with a zero mean and unit variance, and x(i) corresponds to a normalized constellation point of *m*-QAM with a given SNR of *P*. E[·] and Var[·] represent the calculations of the mean and variance, respectively. When the value of *m* is quite large, the calculations of (16) and (17) suffer a noticeable increase in complexity, but do provide the correlation between *C*, *V* and the constellation.

## 3. Practical Application with Retransmission

In this section, we consider the coded modulation retransmission scenario in incremental redundancy (IR) HARQ and the calculations of key parameters with the theoretical formula and a calibration model proposed by Eva C. Song and Guosen Yue [26], which are easy to use and have extremely good accuracy.

When the first segment of a rate-compatible coding scheme with a high-rate code of length *n* fails to be received, the transmitter then sends the redundancy version of coded bits with identical length *n* to the receiving end, resulting in a half-rate code of length 2n for decoding. During retransmission, the modulation order is usually lowered according to the specific modulation and coding scheme (MCS), such as the MCS table in 5G NR, thereby better handling errors and enhancing the robustness of transmission.

In [26], the calculations of *C* and *V* for the parallel complex Gaussian channels with an *m*-QAM input are provided by (15) and (16), respectively. Similarly, we can consider the coded modulation in the retransmission scenario as the receiver simultaneously receiving two equal-length coded blocks from a rate-compatible coding scheme with different modulations $m_{1}$-QAM and $m_{2}$-QAM over the same channel.

Therefore, in this scenario, *C* and *V* in (9) are computed by

(18) $$C=\frac{1}{2}\left(C_{m1}\left(P\right)+C_{m2}\left(P\right)\right)$$

(19) $$V=\frac{1}{2}\left(V_{m1}\left(P\right)+V_{m2}\left(P\right)\right),$$

where $C_{m_{i}}\left(P\right)$ and $V_{m_{i}}\left(P\right),i=1,2$ can get by (16) and (17) on the constellations of $m_{1}$-QAM and $m_{2}$-QAM, respectively. The proof of *C* and *V* is provided in Appendix A.

In terms of practical coding, ref. [26] proposes the following models:

(20) $$\begin{array}{c} R\left(P,n,\epsilon \right)=C\left(P\right)-\Delta C\left(P\right)-\alpha \left(P\right)\sqrt{\frac{V(P)}{n}}Q^{-1}\left(\epsilon \right)+\frac{\log_{2}\left(n\right)}{2n}, \end{array}$$

where C(P) and V(P) can get by (18) and (19), respectively. ΔC(P) refers to the gap between the theoretical capacity and the rate that practical coding can achieve when the blocklength is finite. α(P)≥1 is the correction parameter for the channel dispersion *V*.

**Figure 1:**
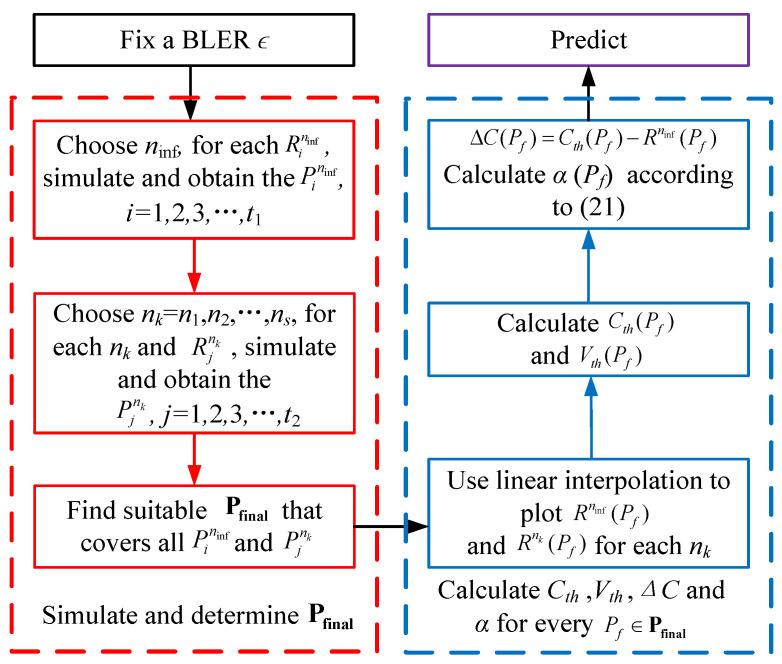
Flowchart of calculation algorithm.

We follow the flowchart shown in Figure 1 to calculate the parameters in (20). Firstly, select a targeting BLER ϵ and a sufficiently long blocklength $n_{\inf}$ as an approximation for infinite blocklength, where a practical rate-compatible coding scheme is employed for the necessary initial simulation, such as LTE-turbo codes and 5G-LDPC codes. Then, for each specific rate $R_{i}^{n_{\inf}},i=1,2,3,...,t_{1}$, obtain the $P_{i}^{n_{\inf}},i=1,2,3,...,t_{1}$ required to achieve the BLER ϵ based on simulation. Next, select several short blocklengths $n_{1},n_{2},...,n_{s}$ for tuning. For each $n_{k},k=1,...,s$ and specific rate $R_{j}^{n_{k}},j=1,2,3,...,t_{2}$, obtain the $P_{j}^{n_{k}},j=1,2,3,...,t_{2}$ required to achieve the BLER ϵ based on simulation.

We simulate to obtain the $P_{i}^{n_{\inf}}$ and $P_{j}^{n_{k}}$ variables using the following method: Given the modulation scheme, code blocklength $n,n=n_{\inf}$ or $n_{k}$, and rate *R*, we vary the values of SNR to obtain a set of data for different SNRs and BLERs $(P_{m}^{n},\epsilon_{m}),m=1,2,3,...$. Then, around the given BLER ϵ, we identify two different BLERs which are the nearest neighbors $\epsilon_{m_{1}}>\epsilon >\epsilon_{m_{2}}$ and perform linear interpolation based on their corresponding SNRs $P_{m_{1}}^{n}$ and $P_{m_{2}}^{n}$ to obtain the SNR $P^{n}$ corresponding to the desired BLER ϵ. The linear interpolation formula is as follows.

(21) $$\epsilon =\frac{\epsilon_{m_{2}}-\epsilon_{m_{1}}}{P_{m_{2}}^{n}-P_{m_{1}}^{n}}\left(P^{n}-P_{m_{1}}^{n}\right)+\epsilon_{m_{1}}.$$

Let the ϵ be the desired BLER; then, we can get the SNR $P^{n}$ by (21).

Next, based on the $P_{i}^{n_{\inf}}$ and $P_{j}^{n_{k}}$ obtained from above, determine a smallest range (or slightly larger) of $P_{\mathrm{final}}$ to cover all the $P_{i}^{n_{\inf}}$ and $P_{j}^{n_{k}}$. For example, if we simulate to obtain $P_{i}^{n_{\inf}}=1,1.5,...,2.5$ (dB) and $P_{j}^{n_{k}}=1.2,1.7,2.2,...,2.9$ (dB), then we can choose $P_{\mathrm{final}}=\left[1,2.9\right]$ (dB). After that, use linear interpolation to connect all the $R_{i}^{n_{\inf}}\left(P_{i}^{n_{\inf}}\right)$ and $R_{j}^{n_{k}}\left(P_{j}^{n_{k}}\right)$ to get $R^{n_{\inf}}\left(P_{f}\right)$ and $R^{n_{k}}\left(P_{f}\right)$ in the range $P_{f}\in P_{\mathrm{final}}$. Then, calculate the theoretical channel capacity $C_{th}\left(P_{f}\right)$ and the theoretical channel dispersion $V_{th}\left(P_{f}\right)$ according to (18) and (19), respectively. Next, calculate $\Delta C\left(P_{f}\right)=C_{th}\left(P_{f}\right)-R^{n_{\inf}}\left(P_{f}\right)$ and for every $P_{f}\in P_{\mathrm{final}}$, find α that minimizes (22) to get $\alpha (P_{f})$.

(22) $$\begin{array}{c} \alpha \left(P_{f}\right)=\underset{\alpha }{\arg\min}\sum_{k=1}^{s}(C_{th}\left(P_{f}\right)-\Delta C\left(P_{f}\right)-\alpha \sqrt{\frac{V_{th}\left(P_{f}\right)}{n_{k}}}Q^{-1}\left(\epsilon \right)+\frac{\log_{2}n_{k}}{2n_{k}}-R^{n_{k}}\left(P_{f}\right){)}^{2} \end{array}$$

Finally, for any given code length *n* and $P_{f}\in P_{\mathrm{final}}$, compute *C* and *V* according to (18) and (19), and obtain ΔC and α from the steps above. Predict *R* by using (20), which is shown in Algorithm 1.
**Algorithm 1:** Calculation algorithm of the model to predict R **Input**: ϵ, $n_{\inf}$, $R_{i}^{n_{\inf}}$, $n_{1},n_{2},...,n_{s}$, $R_{j}^{n_{k}}$ **Output**: *R*1Fix a BLER ϵ2Simulate to get $P_{i}^{n_{\inf}}$ based on $n_{\inf}$, ϵ, $R_{i}^{n_{\inf}},i=i=1,2,3,...,t_{1}$3Simulate to get $P_{j}^{n_{k}}$ based on $n_{k}=n_{1},n_{2},...,n_{s}$, ϵ, $R_{j}^{n_{k}},j=1,2,3,...,t_{2}$4Choose a range $P_{\mathrm{final}}$ that covers all the $P_{i}^{n_{\inf}}$ and $P_{j}^{n_{k}}$5Use linear interpolation to connect all the $R_{i}^{n_{\inf}}\left(P_{i}^{n_{\inf}}\right)$ and $R_{j}^{n_{k}}\left(P_{j}^{n_{k}}\right)$ to get $R^{n_{\inf}}\left(P_{f}\right)$ and $R^{n_{k}}\left(P_{f}\right)$ in the range $P_{f}\in P_{\mathrm{final}}$6Calculate $C_{th}\left(P_{f}\right)$, $V_{th}\left(P_{f}\right)$ for $P_{f}\in P_{\mathrm{final}}$7$\Delta C\left(P_{f}\right)=C_{th}\left(P_{f}\right)-R^{n_{\inf}}\left(P_{f}\right)$ for $P_{f}\in P_{\mathrm{final}}$8For every $P_{f}\in P_{\mathrm{final}}$, calculate α that minimizes (22) to get $\alpha (P_{f})$9**return**$$\begin{array}{c} R=C\left(P_{f}\right)-\Delta C\left(P_{f}\right)-\alpha \left(P_{f}\right)\sqrt{\frac{V(P_{f})}{n}}Q^{-1}\left(\epsilon \right)+\frac{\log_{2}\left(n\right)}{2n} \end{array}$$for every $P_{f}\in P_{\mathrm{final}}$

The above method incorporates some modifications to the method proposed in [26]. When using (22), the SNR required for calculating each α is the same, but the simulated SNR often varies for different selected $n_{k}$ and $R_{j}^{n_{k}}$. Therefore, after obtaining the simulation data points, we select a range $P_{\mathrm{final}}$ to unify the different SNRs obtained from the simulation that required in the formula.

## 4. Numerical Example

In this section, we demonstrate that the proposed model is also applicable to the scenario of retransmission and we analyze the results with different coded modulation combinations. In the following examples, we always use the rate-compatible coding scheme based on 5G-LDPC codes and BP decoding in the transmitter and receiver. Assume that 16-QAM and QPSK are used in two transmissions, respectively, where the coded bits in the first half and the second half of each encoding segment are modulated by 16-QAM and QPSK, respectively.

In our simulations, the rate *R* is computed by

(23) $$R=R_{c}\times \frac{1}{2}\left(\log_{2}\left(m_{1}\right)+\log_{2}\left(m_{2}\right)\right),$$

where $R_{c}$ is the original code rate, $\log_{2}\left(m_{1}\right)$ and $\log_{2}\left(m_{2}\right)$ refer to the modulation orders for the two segments. In this example, an LDPC code with a code rate of $R_{c}=\frac{1}{3}$, 16-${\mathrm{QAM}}_{1st}$$\log_{2}\left(m_{1}\right)=4$, and ${\mathrm{QPSK}}_{2nd}$$\log_{2}\left(m_{2}\right)=2$ are employed in the two transmissions; thus, the rate here is R=1.

Since the number of message bits remains the same after retransmission, the code length becomes twice as long, and the highest code rate of 5G-LDPC codes is $\frac{11}{12}$ in the first transmission. Then, after retransmission with $R_{c}=\frac{11}{24}$, the highest rate here is $R=\frac{11}{24}\times 3=\frac{11}{8}$.

We select $n_{\inf}=7200$ as an approximation for infinite code length, which approaches the maximum length 8448 of information bits in the 5G-LDPC coding scheme, with $n_{1}=\frac{1}{20}n_{\inf}=360$, $n_{2}=\frac{3}{20}n_{\inf}=1080$ for tuning. We choose the code rates like $R_{c}=\frac{1}{3},\frac{7}{20},\frac{11}{30},\frac{5}{12},\frac{9}{20}$ and $\frac{11}{24}$ to make the code with length 360 have an integer number of information bits. Then, we predict the retransmission performance of the coded modulation with n=3600.

**Figure 2:**
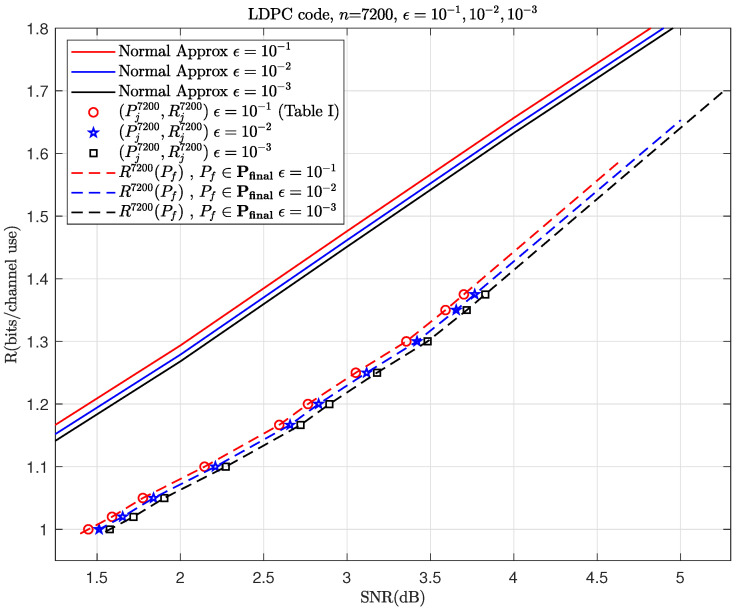
Calculate ΔC for HARQ using 16-${\mathrm{QAM}}_{1st}$ in the 1st transmission and ${\mathrm{QPSK}}_{2nd}$ in the 2nd one with different BLERs, where the red circles, blue stars and black squares correspond to the points for $\epsilon =10^{-1}$ (listed in Table 1), $10^{-2}$ and $10^{-3}$, respectively.

**Figure 3:**
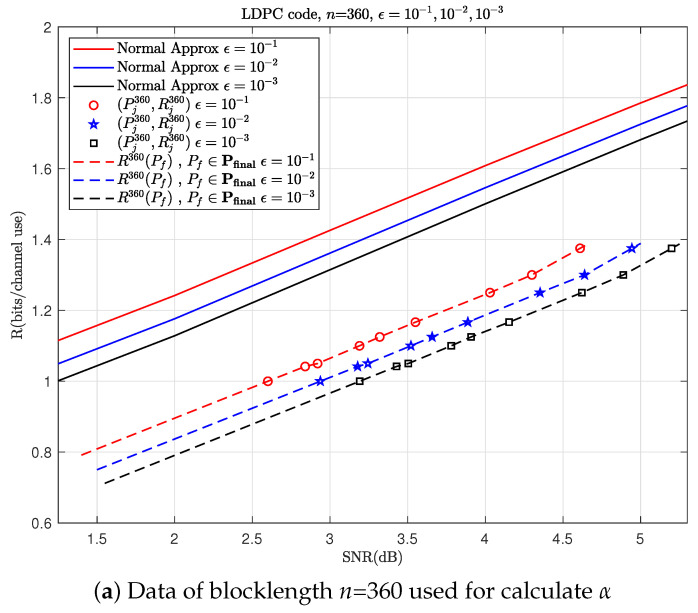

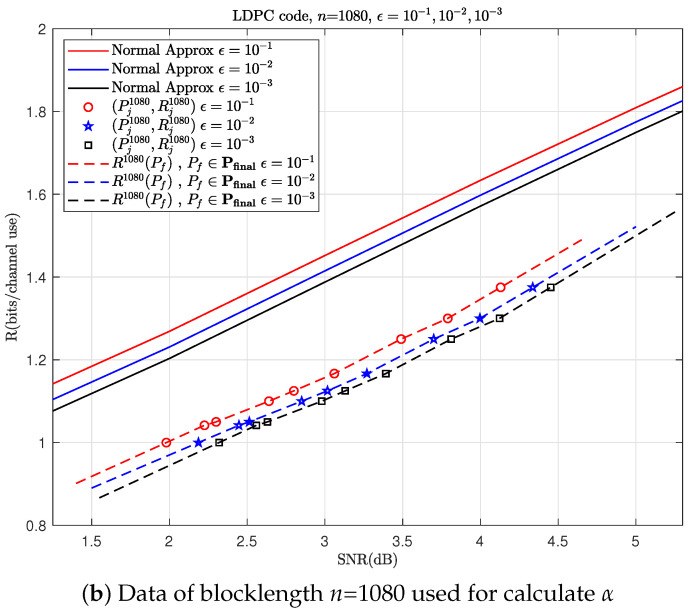
Calculate α for HARQ using 16-${\mathrm{QAM}}_{1st}$ in the 1st transmission and ${\mathrm{QPSK}}_{2nd}$ in the 2nd one with different BLERs, where the red circles, blue stars and black squares correspond to the points for $\epsilon =10^{-1}$ (listed in Table 2), $10^{-2}$ and $10^{-3}$, respectively.

**Figure 4:**
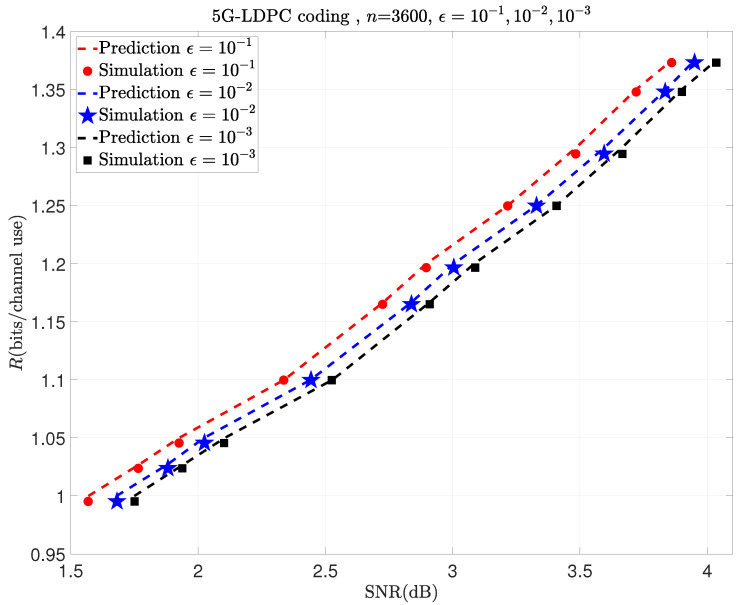
Prediction results of HARQ using 16-${\mathrm{QAM}}_{1st}$ in the 1st transmission and ${\mathrm{QPSK}}_{2nd}$ in the 2nd one with different BLERs.

With this example, let us go through the steps outlined in Algorithm 1. Choose a fixed BLER ϵ=0.1 and then simulate to obtain Figure 2 and Figure 3a,b. In this example, $P_{\mathrm{final}}=\left[1.4,4.65\right]$ (dB) can cover all the simulation points $P_{i}^{n_{\inf}}$ and $P_{j}^{n_{k}}$. Then, Table 1 and Table 2 calculate ΔC and α, respectively. Finally, using (20) and the previously obtained parameters, we can predict *R*. By repeating the steps mentioned above for $\epsilon =10^{-2}$ and $10^{-3}$, we can get the results shown in Figure 4. As shown in Figure 4, the prediction performance of this model is also very good in the retransmission scenario with a moderate blocklength and different modulations.

**Figure 5:**
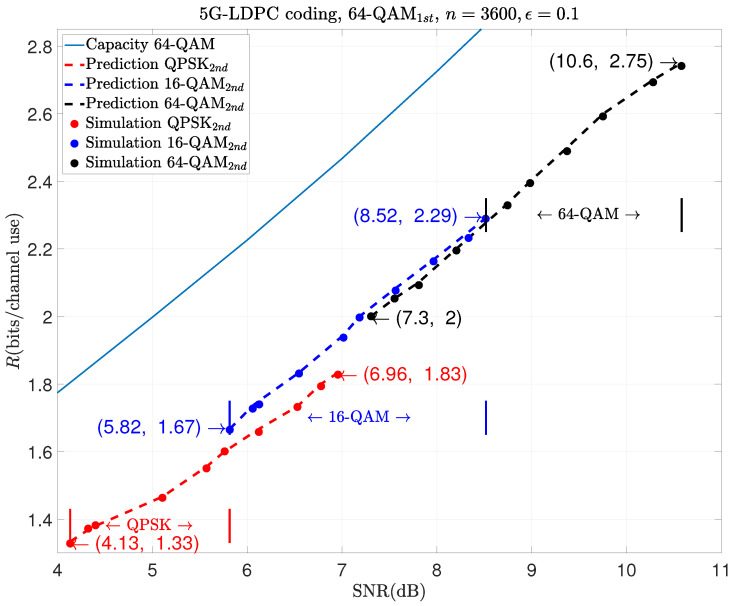
Prediction results of HARQ using 64-${\mathrm{QAM}}_{1st}$ in the 1st transmission and $m_{2}$-${\mathrm{QAM}}_{2nd}$ in the 2nd one.

**Figure 6:**
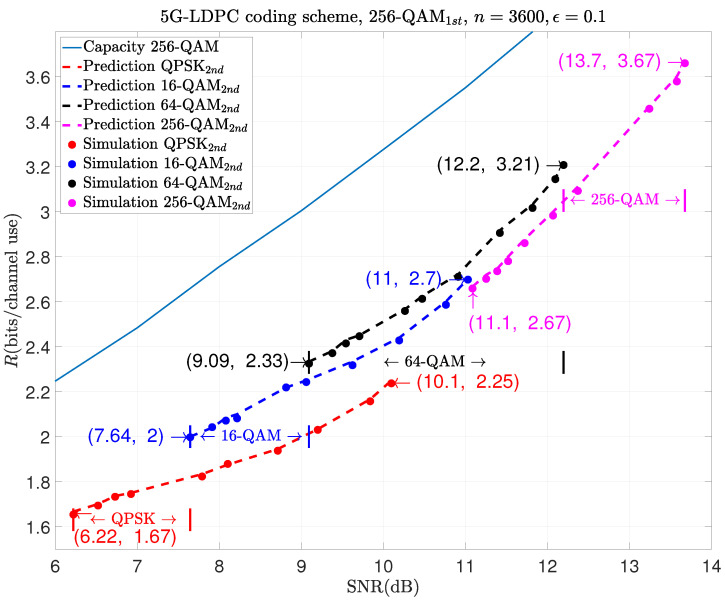
Prediction results of HARQ using 256-${\mathrm{QAM}}_{1st}$ in the 1st transmission and $m_{2}$-${\mathrm{QAM}}_{2nd}$ in the 2nd one.

**Figure 7:**
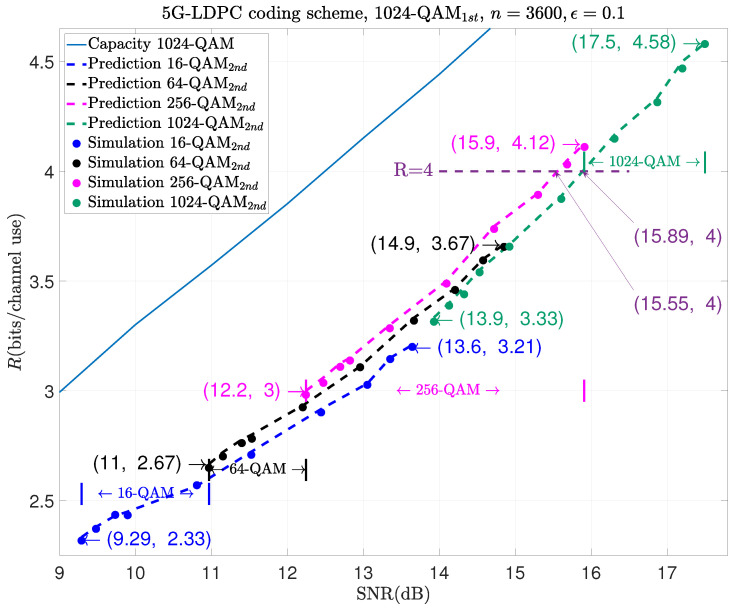
Prediction results of HARQ using 1024-${\mathrm{QAM}}_{1st}$ in the 1st transmission and $m_{2}$-${\mathrm{QAM}}_{2nd}$ in the 2nd one.

To more clearly distinguish between the simulation data points used before prediction and those used to validate the accuracy of the prediction afterward, we plot the simulation data points required before prediction in Figure 2 and Figure 3a,b as hollow points and the simulation data points used for validation after prediction in Figure 4, Figure 5, Figure 6 and Figure 7 as solid points, respectively. The predicted curves in Figure 4, Figure 5, Figure 6 and Figure 7 are derived from the initial simulations, Algorithm 1, as well as the analytical formulas. The simulation points on the predicted curves are obtained by selecting certain SNRs and spectral efficiencies within the interval after predicting the performance and then conducting simulations for verification. The comparisons between the simulated points and the predicted curves show very small discrepancies. When the SNR is the same, the simulation value may be a little lower than the prediction curve.

The calculation environments for simulations and predictions are the same. We use MATLAB 2023b to calculate, use MATLAB’s built-in functions *ldpcEncode* and *ldpcDecode* for encoding and decoding LDPC codes, use MATLAB’s built-in functions *qammod* and *qamdemod* for modulation and demodulation, and we use MATLAB’s built-in functions *awgn* to add noise.

The following examples show the prediction results of moderate-blocklength coded retransmission with other modulation schemes, like 1024-${\mathrm{QAM}}_{1st}$ and 256-${\mathrm{QAM}}_{2nd}$, in the first and the second transmissions. They are shown in Figure 5, Figure 6 and Figure 7, respectively.

As shown in these figures, different combinations of modulation schemes can cover different ranges of rates. In Figure 5, we can see that the combination of 64-QAM and QPSK covers the rate range from 1.33 to 1.83 in the SNR from 4.13 dB to 6.96 dB; that of 64-QAM and 16-QAM covers the rate range from 1.67 to 2.29 in the SNR from 5.82 dB to 8.52 dB; and 64-QAM combined with 64-QAM covers the rate range from 2 to 2.75 in the SNR from 7.3 dB to 10.6 dB.

The results in Figure 6 show that if 256-QAM is used for the initial transmission and if the aim for a retransmission is to achieve the rate range between 2 and 2.25, we can use QPSK or 16-QAM for the retransmission. In this case, the required SNR range is between 7.64 dB and 10.1 dB. Similarly, if 16-QAM or 64-QAM is used for the retransmission, the corresponding SNR range is between 9.09 dB and 11 dB, resulting in a rate range of 2.33 to 2.7. If 256-QAM is still used during retransmission, it will cover a rate range from 2.67 to 3.67 in the SNR from 11.1 dB to 13.7 dB.

However, we can also see in these three figures that using the same modulation scheme during retransmission as before results in poorer performance. For example, in Figure 7, to achieve the same rate like R=4, the combination of 1024-QAM and 1024-QAM performs about 0.34 dB poorer than that of 1024-QAM and 256-QAM.

The results above can guide us in selecting different modulation schemes based on varying data rate requirements during retransmission. For example, as illustrated in Figure 7, when using 1024-QAM for the initial transmission, if the rate is between 2.33 and 2.67, it would be better to use 16-QAM for retransmission. This is because the minimum code rate of LDPC code is 1/3, and the minimum rate of 1024-QAM combined with 64-QAM is $R=\frac{1}{3}\times \frac{1}{2}\times \left(10+6\right)=2.67$. Similarly, if the rate is between 3 and 4.12, it would be better to use 256-QAM for retransmission because the maximum code rate after retransmission is $R_{c}=\frac{11}{12}\times \frac{1}{2}=\frac{11}{24}$, and the maximum rate of 1024-QAM combined with 256-QAM is $R=\frac{11}{24}\times \frac{1}{2}\times \left(10+8\right)=4.12$. If the rate is greater than 4.12, we only use 1024-QAM to retransmit. Similar conclusions can be drawn for 64-QAM and 256-QAM in Figure 5 and Figure 6.

## 5. Efficiency Analysis

**Figure 8:**
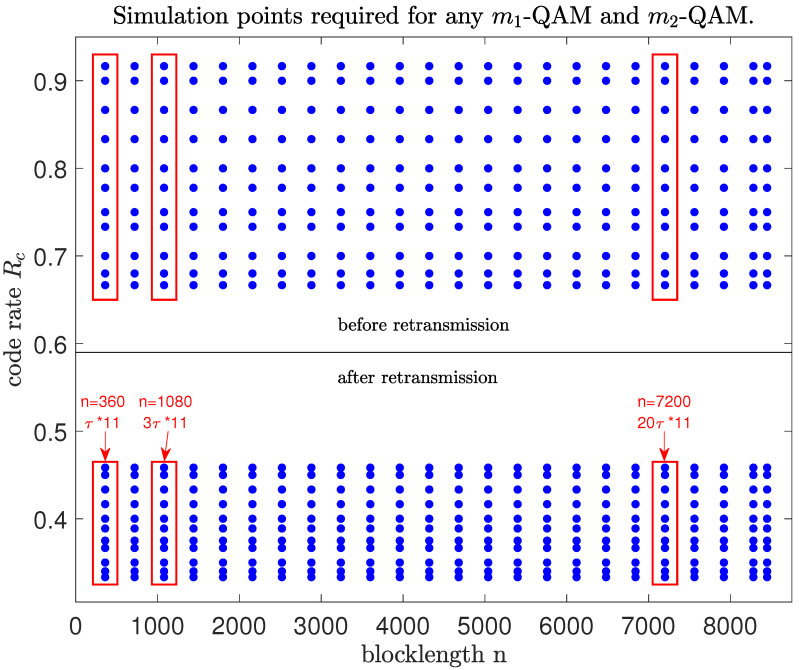
The link settings required for system-level simulations with different code rates and blocklengths, where $m_{1}$-QAM and $m_{2}$-QAM are used in the first transmission and second transmission, respectively.

Figure 8 takes the MCS of 5G NR with the BG1 matrix as an example, where we select a set of coding parameters with blocklengths ranging from n=360 (Z=18) to the maximum length of n=8448 (Z=384). The ranges of code rates, respectively, cover [$\frac{2}{3}$, $\frac{11}{12}$] and [$\frac{1}{3}$, $\frac{11}{24}$] in the first transmission and retransmission with a total $n_{R_{c}}=11$ different code rates. Increasing the value of $n_{R_{c}}$ can further fine-tune the prediction accuracy. For the system-level simulations that are crucial for the design of 5G networks, it is generally required to obtain the link-level BLER performance metrics for all data points in Figure 8 through simulations. Then, for the specific link settings of code rates and blocklengths, the BLER performances can be directly obtained via linear interpolation with the nearby data points. For future mobile communication systems, with wider ranges of code rates and blocklengths and lower BLER targets, the performance evaluations for link-level simulations with multiple retransmissions will significantly increase the computational complexity. Our proposed performance prediction scheme can effectively reduce the computational load while ensuring evaluation accuracy.

In the above example of MCSs shown in Figure 8, once the $m_{1}$-QAM, $m_{2}$-QAM and BLER are determined before or after retransmission, we only need to simulate all the rate data for three sets of blocklengths (the blue points in three red rectangle boxes in Figure 8) to predict the rates for any other blocklength (other blue points in Figure 8). Hence, when the BLERs and SNRs for the coded modulation combinations with all the different blocklengths and code rates are required for system-level simulations, using our algorithm can significantly improve the efficiency of performance evaluations. As shown in Figure 8, assuming that a single testing of BLER ϵ evaluation for a code with n=360 requires a time of τ, then obtaining one set of data requires a time of τ×11 for all code rates considered before or after retransmission. As the blocklength increases, the simulation time will also increase linearly, which means that the simulation testings of performance evaluation for the codes with n=1080 and n=7200 require a computation time of 3τ and 20τ, respectively. Therefore, the total simulation time required to obtain all the data needed for the performance prediction of MCSs with $m_{1}$-QAM and $m_{2}$-QAM is (τ+3τ+20τ)×11=264τ. Since the time required for the calculations of ΔC and α is negligible compared to that of simulation tests for performance evaluations, the total computational complexity needed to complete the entire prediction can be approximately evaluated by 264τ. Then, if we need the rate data of codes from n=360 to 8448 according to all the lifting values of 5G-LDPC codes shown in Figure 8, the total computational complexity required for a brute-force Monte Carlo simulation should be about 3300τ, which is clearly greater than 264τ. Since each modulation combination for retransmission requires a separate simulation, this algorithm can significantly reduce the computational complexity when a large amount of SNR-R relationship data corresponding to various blocklengths are needed, given a specific BLER ϵ.

## 6. Conclusions

In this paper, we have reviewed the theoretical prediction formulas for the performance of finite-length coding and their correction models. Through simulation, we validated the good adaptability of the correction model to the retransmission scenarios. To make this model more general, we introduced a range $P_{\mathrm{final}}$ to unify the different SNRs. Based on the simulation results, we can choose the modulation method for the second transmission according to different bit rate requirements. It is also evident that if the same modulation method is employed in the second transmission as before, its performance is not as effective as some methods involving a reduction in the modulation order during retransmission.

## Figures and Tables

**Table 1:** Calculate ΔC for HARQ using 16-${\mathrm{QAM}}_{1st}$ in the 1st transmission and ${\mathrm{QPSK}}_{2nd}$ in the 2nd one, $n_{\inf}$ = 7200, ϵ = $10^{-1}$. Here, some points in $P_{\mathrm{final}}$ are shown.

$P_{f}\in P_{\mathrm{final}}$ (dB)	$C_{\mathrm{th}}$	$R^{7200}$	ΔC
1.45	1.2	1	0.2
1.775	1.26	1.05	0.21
2.145	1.32	1.1	0.22
2.593	1.4	1.167	0.233
2.765	1.43	1.2	0.23
3.052	1.49	1.25	0.24
3.355	1.54	1.3	0.24
3.59	1.58	1.35	0.23
3.7	1.61	1.375	0.235

**Table 2:** Calculate α for HARQ using 16-${\mathrm{QAM}}_{1st}$ in the 1st transmission and ${\mathrm{QPSK}}_{2nd}$ in the 2nd one, $n_{1}$ = 360, $n_{2}$ = 1080, ϵ = $10^{-1}$. Here, some points in $P_{\mathrm{final}}$ are shown.

$P_{f}\in P_{\mathrm{final}}$ (dB)	$R^{360}$	$R^{1080}$	$C_{\mathrm{th}}$	$V_{\mathrm{th}}$	ΔC	α
2.6	1	1.09	1.42	1.383	0.23	1
2.75	1.026	1.117	1.446	1.375	0.23	1
2.9	1.048	1.141	1.475	1.366	0.23	1
3.05	1.074	1.165	1.501	1.356	0.24	1.1849
3.2	1.102	1.194	1.529	1.344	0.24	1.6132
3.35	1.13	1.223	1.556	1.332	0.24	2.1820
3.5	1.157	1.251	1.583	1.319	0.235	2.5282

## Data Availability

Data are contained within the article.

## Appendix A

Random variables and its realization are denoted by capital letters *X* and its lower case *x*, respectively. The boldface letter denotes a vector. A sequence $x_{1},...,x_{n}$ is denoted by $x^{n}$. A sequence $x^{n}$ partitioned into 2 blocks with equal blocklength $n_{1}=n_{2}=\frac{n}{2}$ is denoted by $x^{n}=\left[x_{1},x_{2}\right]$, where *n* is the blocklength of the code, and the *i*th element of vector $x_{1},x_{2}$ is denoted by $x_{1,i},x_{2,i}$. The total variational distance between two distributions *D* and *Q* is denoted by ${∥D-Q∥}_{TV}$. The Radon-Nikodym derivative of *D* w.r.t. *Q* is denoted by $\frac{dD}{dQ}$. Expectations and variances taken w.r.t. a distribution *D* are indicated by $E_{D}\left[\cdot \right]$ and ${\mathrm{Var}}_{D}\left[\cdot \right]$, respectively.

Let $\left(Q_{X}\left(x\left(1\right)\right),...,Q_{X}\left(x\left(m\right)\right)\right)$ denote an input distribution, where x(i)’s are the *m*-QAM constellation points with average power Pw. Denote by ${\hat{D}}_{X}$ the composition $\left({\hat{D}}_{X}\left(x\left(1\right)\right),...,{\hat{D}}_{X}\left(x\left(m\right)\right)\right)=\left({\hat{Q}}_{X}\left(x\left(1\right)\right),...,{\hat{Q}}_{X}\left(x\left(m\right)\right)\right)$, if $nQ_{X}\left(x\right)$ is an integer for all *x*, or otherwise, the type closest to the $Q_{X}$ in total variational distance, in which the greater probabilities are assigned first to the indices i∈{1,...,m} whose corresponding constellation symbols x(i) have individual power equal to Pw (if available), then to indices whose corresponding symbols have individual power less than Pw, and finally to those with corresponding individual power greater than Pw.

In the retransmission scenario, the 2 segments are effectively transmitted over the same BIAWGN channel, hence:

(A1) $$Y_{k}=X_{k}+W_{k},$$

where $W_{k}∼CN\left(0_{n_{k}},I_{n_{k}}\sigma_{k}^{2}\right),k=1,2$, $\sigma_{1}=\sigma_{2}=\sigma$, and $I_{n_{k}}$ is an identity matrix. Therefore, the SNR is

(A2) $$P=\frac{Pw}{\sigma }.$$

The proof of the channel capacity *C* and the channel dispersion *V* in the retransmission scenario is an application of Theorem 2 from [25], which is stated as follows.

**Theorem A1.** 
*For a channel $D_{Y^{n}|X^{n}}\left(y^{n}|n^{n}\right)$ any input distribution $D_{X^{n}}$, and any output distributuion $Q_{Y^{n}}$, there exists a code with M codewords in $F_{n}$ and average probability of error satisfying*

(A3)
$$\epsilon \leq D_{X^{n}}D_{Y^{n}|X^{n}}\left[\tilde{i}\left(X^{n};Y^{n}\right)\leq \log_{2}\gamma_{n}\right]+L_{n}MD_{X^{n}}Q_{Y^{n}}\left[\tilde{i}\left(X^{n};Y^{n}\right)>\log_{2}\gamma_{n}\right]+D_{X^{n}}\left[X^{n}\notin F_{n}\right]$$

*where*

(A4)
$$\tilde{i}\left(X^{n};Y^{n}\right)=\log_{2}\frac{D_{Y^{n}|X^{n}}\left(Y^{n}|X^{n}\right)}{Q_{Y^{n}}\left(Y^{n}\right)}$$

*and the coefficient $L_{n}$ is defined as*

(A5)
$$L_{n}\overset{\Delta }{=}\underset{y^{n}\in Y^{n}}{\sup}\frac{dD_{Y^{n}}\left(y^{n}\right)}{dQ_{Y^{n}}\left(y^{n}\right)}$$

*and $\gamma_{n}$ is an arbitary positive threshold whose optimal choice to give the highest rates is $\gamma_{n}=L_{n}M$.*

This proof follows similar steps as the proof for the single AWGN channel in [25] with the modification of taking into account the retransmission scenario. For the 2 segments of length $n_{k}=\frac{n}{2},k=1,2$, denote by ${\hat{D}}_{X}^{\left(k\right)}$ the composition of segment *k* and $T_{n_{k}}\left({\hat{D}}_{X}^{\left(k\right)}\right)$ the corresponding type class. For the different modulation schemes $m_{1}$-QAM and $m_{2}$-QAM of the 2 segments. We fix

(A6) $$Q_{X}\left(x(i)\right)=\frac{1}{m_{k}},i=1,...,m_{k},k=1,2.$$

Under this construction, it can be shown that

(A7) $${∥{\hat{D}}_{X}^{\left(k\right)}-Q_{X}∥}_{TV}\leq O\left(\frac{1}{n_{k}}\right),$$

and

(A8) $$E_{{\hat{D}}_{X}^{\left(k\right)}}\left[{∥X∥}^{2}\right]\leq Pw.$$

We choose the input distribution $D_{X^{n}}$ as the following:

(A9) $$D_{X^{n}}\left(x^{n}\right)=\prod_{k=1}^{2}\frac{\left\{x_{k}\in T_{n_{k}}\left({\hat{D}}_{X}^{\left(k\right)}\right)\right\}}{\left(n_{k}{\hat{D}}_{X}^{\left(k\right)}\left(x(1)\right),...,n_{k}{\hat{D}}_{X}^{\left(k\right)}\left(x(m_{k})\right)\right)}.$$

Let

(A10) $$F_{n}=\left\{x^{n}:{∥x_{k}∥}^{2}\leq n_{k}Pw,k=1,2\right\}.$$

It can be verified that the input distribution from (A7) satisfies the maximal power constraint ${∥x_{k}∥}^{2}\leq n_{k}Pw$. Hence,

(A11) $$D_{X^{n}}\left[X^{n}\notin F_{n}\right]=0.$$

The output distribution induced by the input distribution $D_{X^{n}}$ can be written as

(A12) $$D_{Y^{n}}\left(y^{n}\right)=\sum_{x^{n}\in X^{n}}D_{X^{n}}\left(x^{n}\right)D_{Y^{n}|X^{n}}\left(y^{n}|x^{n}\right)=\prod_{k=1}^{2}\sum_{x_{k}\in T_{n_{k}}\left({\hat{P}}_{X}^{\left(k\right)}\right)}\Gamma_{k}D_{Y_{k}|X_{k}}\left(y_{k}|x_{k}\right),$$

where

(A13) $$\Gamma_{k}=\frac{1}{\left(\begin{array}{c} n_{k}\\ n_{k}{\hat{D}}_{X}^{\left(k\right)}\left(x(1)\right),...,n_{k}{\hat{D}}_{X}^{\left(k\right)}\left(x(m_{k})\right) \end{array}\right)}$$

and

(A14) $$D_{Y_{k}|X_{k}}\left(y_{k}|x_{k}\right)=\prod_{t=1}^{n_{k}}D_{Y|X}^{\left(k\right)}\left(y_{t}|x_{t}\right)$$

and $D_{Y|X}^{\left(k\right)}$ indicates the channel experienced by segment k,k=1,2.

Next, we choose the following auxiliary distributions:

(A15) $$Q_{Y}^{\left(k\right)}\left(y\right)=\sum_{i=1}^{m_{k}}Q_{X}\left(x(i)\right)D_{Y|X}^{\left(k\right)}\left(y|x(i)\right),k=1,2$$

(A16) $$Q_{Y^{n}}\left(y^{n}\right)=\prod_{k=1}^{2}\prod_{t=1}^{n_{k}}Q_{Y}^{\left(k\right)}\left(y_{k,t}\right)$$

where $Q_{X}$ is given in (A6).

It can be shown by applying Proposition 3 of [25] to (A12) and (A16), we have

(A17) $$\frac{dP_{Y^{n}}\left(y^{n}\right)}{dQ_{Y^{n}}\left(y^{n}\right)}\leq L_{n}\overset{\Delta }{=}\prod_{k=1}^{2}c_{k}\left(m_{k}\right)n_{k}^{\frac{m_{k}-1}{2}}$$

for sufficiently large $n_{k}$’s, where $c_{k}\left(m_{k}\right)$’s are positive constants that depend only on the constellation size *m*.

We now apply Theorem A1 to the distributions defined above. Let $\gamma_{n}=L_{n}M$. For the first term in (A3), it can be verified that

(A18) $$\frac{1}{n}\tilde{i}\left(x^{n};Y^{n}\right)=-\frac{1}{n}\sum_{k=1}^{2}\sum_{t=1}^{n_{k}}\log_{2}\left(\sum_{j=1}^{m_{k}}Q_{X}\left(x(j)\right)e^{\frac{{∥W_{k,t}∥}^{2}-{∥x_{k,t}+W_{k,t}-x\left(j\right)∥}^{2}}{\sigma^{2}}}\right).$$

Since {$W_{k,t}$}’s are independent, we can invoke the Berry-Esseen Theorem on $\frac{1}{n}\tilde{i}\left(x^{n};Y^{n}\right)$. Using (A7), the mean can be verified to be

(A19) $$E_{D_{Y^{n}|X^{n}=x^{n}}}\left[\frac{1}{n}\tilde{i}\left(x^{n};Y^{n}\right)\right]=\frac{1}{2}\left(C_{m1}\left(P\right)+C_{m2}\left(P\right)\right)+O\left(\frac{1}{n}\right)=C+O\left(\frac{1}{n}\right).$$

Similarly, the variance can be verified to be

(A20) $$\mathrm{Va}r_{D_{Y^{n}|X^{n}=x^{n}}}\left[\frac{1}{n}\tilde{i}\left(x^{n};Y^{n}\right)\right]=\frac{1}{n}\left(\frac{1}{2}\left(V_{m1}\left(P\right)+V_{m2}\left(P\right)\right)\right)+O\left(\frac{1}{n}\right)=\frac{V}{n}+O\left(\frac{1}{n}\right).$$

Applying the Berry-Esseen Theorem on $\frac{1}{n}\tilde{i}\left(x^{n};Y^{n}\right)$ yields

(A21) $$D_{Y^{n}|X^{n}}\left[\frac{1}{n}\tilde{i}\left(x^{n};Y^{n}\right)\leq \frac{\log_{2}\left(L_{n}M\right)}{n}\right]\leq Q\left(\frac{C-\frac{\log_{2}\left(L_{n}M\right)}{n}}{\sqrt{\frac{V}{n}}}\right)+\frac{B_{1}}{\sqrt{n}},$$

where $B_{1}$ is some positive constant. Consequently, by averaging over the input sequences, the first term from (A3) can be bounded by

(A22) $$D_{X^{n}}D_{Y^{n}|X^{n}}\left[\frac{1}{n}\tilde{i}\left(X^{n};Y^{n}\right)\leq \frac{\log_{2}\left(L_{n}M\right)}{n}\right]\leq Q\left(\frac{C-\frac{\log_{2}\left(L_{n}M\right)}{n}}{\sqrt{\frac{V}{n}}}\right)+\frac{B_{1}}{\sqrt{n}}.$$

For the second term in (A3), observe that under the conditional distribution $D_{Y^{n}|X^{n}=x^{n}}$, $\frac{1}{n}\tilde{i}\left(x^{n};Y^{n}\right)$ is a summation of independent random variables with positive variance and finite third absolute moment. Therefore, we can apply the refined large deviation result of Lemma 47 in [24]

(A23) $$Q_{Y^{n}}\left[\tilde{i}\left(x^{n};Y^{n}\right)>\log_{2}\gamma_{n}\right]=E\left[e^{-\tilde{i}\left(x^{n};Y^{n}\right)}\left\{\tilde{i}\left(x^{n};Y^{n}\right)>\log_{2}\left(L_{n}M\right)\right\}\right]\leq \frac{B_{2}}{\sqrt{n}}{\left(L_{n}M\right)}^{-1},$$

where the expectation is taken with respect to $D_{Y^{n}|X^{n}=x^{n}}$ and $B_{2}$ is a positive constant. Therefore, the second term from (A3) can be bounded as

(A24) $$L_{n}MD_{X^{n}}Q_{Y^{n}}\left[\tilde{i}\left(X^{n};Y^{n}\right)>\log_{2}\left(L_{n}M\right)\right]\leq \frac{B_{2}}{\sqrt{n}}.$$

Finally, combining (A22), (A24) and (A11), yields

(A25) $$\epsilon \leq Q\left(\frac{\frac{C-\log_{2}\left(L_{n}M\right)}{n}}{\sqrt{\frac{V}{n}}}\right)+\frac{B}{\sqrt{n}},$$

where $B=B_{1}+B_{2}$. Rearranging (A25) and with a bit of analysis yields the result of *C* and *V* in retransmission scenario for (9).
